# Metachronous Bilateral Ruptured True Deep Femoral Artery Aneurysms: A Case Report

**DOI:** 10.3400/avd.cr.26-00070

**Published:** 2026-07-23

**Authors:** Takehiro Suzuki, Naoki Toya, Yuri Murakami, Takao Ohki

**Affiliations:** 1 Department of Surgery, Division of Vascular Surgery, The Jikei University Kashiwa Hospital, Kashiwa, Chiba, Japan; 2 Department of Surgery, Division of Vascular Surgery, The Jikei University School of Medicine, Tokyo, Japan

**Keywords:** deep femoral artery aneurysm, metachronous, bilateral, rupture

## Abstract

Deep femoral artery aneurysm (DFAA) is rare, and metachronous bilateral occurrence is extremely rare. An 83-year-old man presented with right thigh pain and swelling. He had undergone left DFAA repair at another hospital 13 years earlier, with no contralateral lesion at that time. CT revealed a ruptured right DFAA with hematoma and contrast extravasation. Emergency surgery was performed, and the proximal DFA was ligated without reconstruction due to distal occlusion. The postoperative course was uneventful. Ligation alone may be feasible in selected cases, and long-term follow-up is essential.

## Introduction

Deep femoral artery aneurysm (DFAA) is an extremely rare condition, accounting for approximately 0.5% of all peripheral arterial aneurysms^1,2)^ and 1%–2.6% of femoral artery aneurysms.^3)^ Bilateral involvement is even rarer, reported in approximately 5% of cases.^4)^ Metachronous bilateral involvement appears to be extremely rare. Here, we report a rare case of metachronous bilateral ruptured DFAA occurring over a 13-year interval.

## Case Report

An 83-year-old man with a history of diabetes mellitus, dyslipidemia, and percutaneous coronary intervention (PCI) was referred to our hospital. Thirteen years earlier, he had undergone an aneurysmectomy with ligation of both the inflow and outflow of the left deep femoral artery (DFA) for a left DFAA at another institution. At that time, contrast-enhanced computed tomography demonstrated no aneurysmal change in the right DFA, with no evidence of atherosclerosis or calcification (**Fig. 1**).

**Fig. 1:**
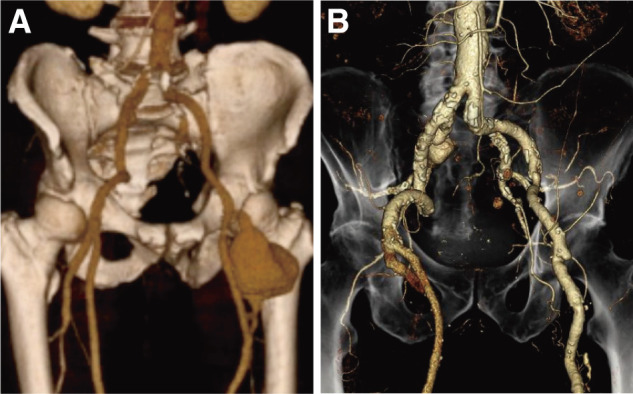
(**A**) Contrast-enhanced CT obtained 13 years before the current presentation, demonstrating a left deep femoral artery aneurysm. No aneurysmal change was identified in the contralateral right deep femoral artery at that time. (**B**) Contrast-enhanced CT at the current presentation, demonstrating a ruptured right deep femoral artery aneurysm.

Approximately 2 months before presentation, the patient noticed pain and subcutaneous ecchymosis in the right thigh and was initially followed conservatively. Because the hematoma gradually enlarged, he was referred to our hospital. Laboratory examination revealed a white blood cell count of 6400/µL and a C-reactive protein level of 3.48 mg/dL. Blood cultures were negative. Contrast-enhanced computed tomography revealed a right DFAA with a giant hematoma measuring 100 × 150 mm extending outside the vascular wall, accompanied by contrast enhancement within the hematoma (**Fig. 2**). Both the femoral vein and the external iliac vein were patent, and no deep vein thrombosis (DVT) was identified. The patient was diagnosed with a ruptured right DFAA, and emergency surgery was performed.

**Fig. 2:**
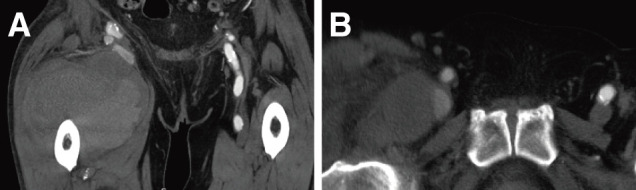
(**A**) Coronal image demonstrating a right DFA aneurysm with a large surrounding hematoma and contrast extravasation. (**B**) Axial image demonstrating a right DFA aneurysm with a large surrounding hematoma. DFA: deep femoral artery aneurysm

A longitudinal incision of approximately 8 cm was made in the right groin, and the common femoral artery (CFA), superficial femoral artery (SFA), and DFA were exposed. Intraoperative angiography via the CFA demonstrated pre-existing occlusion of the distal DFA, with contrast extravasation into the surrounding tissue near the occluded segment (**Fig. 3**). Although the DFAA was large, distal occlusion of the DFA indicated that vascular reconstruction was unnecessary, and the aneurysm was considered amenable to treatment with proximal ligation alone.

**Fig. 3:**
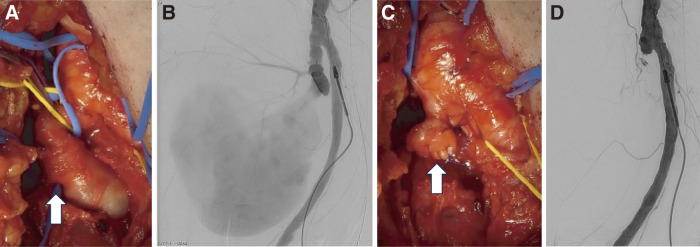
Intraoperative findings and angiography demonstrated the following findings. (**A**) Surgical exposure of the CFA, SFA, and DFA (white arrow) through a longitudinal groin incision. (**B**) Angiography via the CFA demonstrating pre-existing occlusion of the distal DFA and contrast extravasation from the aneurysmal segment. (**C**) Intraoperative view after ligation of the proximal DFA (white arrow). (**D**) Completion angiography confirming complete occlusion of the DFA and disappearance of contrast extravasation. CFA: common femoral artery; SFA: superficial femoral artery; DFA: deep femoral artery

Without extending the skin incision, the proximal DFA was clamped and ligated. The aneurysm was opened, and the aneurysmal contents and wall were resected as much as possible to decompress the hematoma. Angiography demonstrated a focal contrast extravasation consistent with aneurysmal rupture; however, no definite perforation was identified during surgery. As the DFAA was not circumferentially exposed, precise localization of the rupture site was not possible. No backflow from the distal DFA was observed. Repeat angiography via the CFA confirmed complete occlusion of the DFA and disappearance of contrast extravasation, and the procedure was completed (**Fig. 3**). There were no clinical findings suggestive of systemic infection. Histopathological findings are shown in **Fig. 4**.

**Fig. 4:**
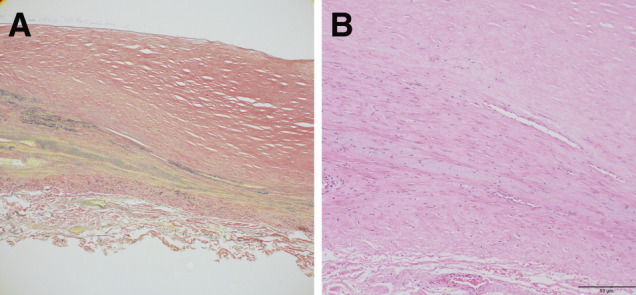
Histopathological findings of the resected deep femoral artery aneurysm. (**A**) Low-power view demonstrating preservation of the 3-layer arterial structure, consistent with a true aneurysm. (**B**) Higher-power view showing atherosclerotic intimal thickening without significant inflammatory cell infiltration.

To further reduce the hematoma volume, ultrasound-guided percutaneous aspiration was performed, and approximately 100 mL of serous yellowish fluid was aspirated. Bacterial cultures were negative, and the aspirated fluid was considered to represent an organized hematoma. The postoperative course was uneventful, and the patient was discharged home ambulatory on postoperative day 13.

During outpatient follow-up, a wound lymphocele and DVT of the femoral vein, presumably caused by compression from the residual hematoma, were identified. The lymphocele resolved with conservative management, and the DVT improved with oral direct anticoagulant therapy. Computed tomography at 30 days postoperatively demonstrated regression of the hematoma without apparent neurological deficits. Follow-up computed tomography at 1 year showed further reduction of the hematoma.

A resected segment of the ligated DFA was submitted for histopathological examination. Histological analysis revealed preservation of the 3-layer arterial structure with atherosclerotic changes in the intima and no marked inflammatory findings, consistent with a true aneurysm.

## Discussion

DFAA predominantly affects men^2)^ and is more commonly reported as a pseudoaneurysm,^5)^ whereas true aneurysms are relatively rare. In the present case, histological analysis revealed preservation of the 3-layer arterial structure with atherosclerotic changes in the intima and no marked inflammatory findings, findings that are consistent with a true aneurysm. DFAA is frequently associated with aneurysmal disease in other arterial territories, with a reported incidence of concomitant aneurysms ranging from 45% to 81%.^3)^ In the present case, a right internal iliac artery aneurysm was also identified.

DFAA is considered less prone to aneurysmal degeneration because of several anatomical factors, including relative resistance to atherosclerotic change, a lower elastic fiber content compared with the femoral artery, and confinement by the surrounding adductor muscles.^6,7)^ Owing to its deep anatomical location, DFAA often remains asymptomatic even after aneurysmal enlargement, and it has been reported that 40.9% of unruptured DFAAs are asymptomatic.^7)^ When symptoms occur, patients may present with painful pulsatile thigh swelling, limb ischemia, ecchymosis, gait disturbance, or sensory impairment.^7)^ Nakai et al. reported a case of DFAA complicated by DVT and pulmonary embolism caused by compression of the deep veins.^8)^ In the present case, DVT of the femoral vein was also observed, presumably due to compression by the giant hematoma.

Once DFAA develops, it tends to enlarge unnoticed and carries a substantial risk of rupture. The lifetime rupture rate has been reported to be approximately 30%–45%.^8,9)^ Although no definitive criteria for surgical intervention have been established, several reports suggest that a diameter of 20 mm or greater is a reasonable threshold for elective repair.^3,10)^ In addition, rapid aneurysmal growth, the presence of symptoms, or rupture are generally considered indications for intervention.^10)^

When treating DFAA, vascular reconstruction is desirable whenever feasible. However, in ruptured cases, anatomical disruption may make reconstruction difficult, and aneurysm ligation may be an alternative option. In patients with concomitant peripheral arterial disease, the profunda femoris artery can serve as an important collateral pathway, and aneurysm resection with ligation may potentially exacerbate limb ischemia; therefore, the safety of ligation alone remains uncertain.^3)^ Posner et al. reported a case of true DFAA treated with ligation and aneurysm decompression alone but emphasized that vascular reconstruction should be considered in patients with non-palpable distal pulses.^4)^ Surgical reconstruction using autologous vein grafts^11)^ or expanded polytetrafluoroethylene grafts has also been reported.^12)^

In recent years, endovascular treatment for DFAA has been increasingly reported. Streck et al. described successful exclusion and revascularization of a 2.9-cm true DFAA using a covered stent.^13)^ Saha et al. reported the first case of endovascular stent graft placement for a ruptured DFAA in an elderly patient, with a favorable postoperative course.^14)^ Both reports suggested that endovascular treatment may be effective in patients with multiple comorbidities.^13,14)^ Okugi et al. reported a case of bilateral DFAA treated with a hybrid approach, combining plug embolization and ligation on one side with stent graft placement on the contralateral side.^15)^ Because DFAA is a rare disease, no studies have directly compared surgical and endovascular treatments, and further accumulation and analysis of cases are required. In the present case, open surgery was selected because the patient presented with rupture associated with a large compressive hematoma requiring immediate decompression. Surgical treatment allowed both rapid hemorrhage control and evacuation of the hematoma. Furthermore, the distal profunda femoris artery was already occluded, making proximal ligation a reasonable treatment strategy. Although endovascular treatment offers a less invasive alternative and may be particularly useful in elderly or high-risk patients, it does not permit evacuation of a large hematoma and may be associated with persistent mass effect. In cases with persistent back bleeding from distal branches, additional distal ligation, direct branch closure within the aneurysm sac, or adjunctive endovascular embolization may be required to achieve complete hemostasis.

In addition to rupture, DFAA may also present with thrombosis or distal embolization, resulting in acute or chronic limb ischemia. Therefore, timely diagnosis and treatment are important even in non-ruptured cases. Although several reports of bilateral DFAA have been published, all previously reported cases were synchronous.^15–17)^ Metachronous bilateral DFAA appears to be extremely rare. In this patient, the distal profunda femoris artery was already occluded, and treatment with ligation and aneurysm decompression alone resulted in a favorable outcome. This case suggests that long-term imaging follow-up is necessary even after treatment of unilateral DFAA, as contralateral aneurysms may develop metachronously.

## Conclusion

We report a rare case of metachronous bilateral ruptured DFAA occurring after contralateral treatment. In selected cases with distal profunda femoris artery occlusion, ligation alone may be a feasible treatment option. Long-term imaging surveillance is important, even after treatment of unilateral DFAA.
